# Electrophysiological Correlates of Familial Dyslexia in Two Siblings: A Case Report

**DOI:** 10.3390/diagnostics16172734

**Published:** 2026-08-26

**Authors:** Milaine Dominici Sanfins, Diego Lourenço dos Santos Silva, Caroline Donadon, Piotr Henryk Skarzynski, Claudia Berlim de Mello, Aparecido J. Couto Soares

**Affiliations:** 1 Department of Speech-Hearing-Language, Universidade Federal de São Paulo, São Paulo 04044-020, SP, Brazilajcsoares@unifesp.br (A.J.C.S.); 2 Department of Teleaudiology and Screening, World Hearing Center, Institute of Physiology and Pathology of Hearing, 05-830 Warsaw, Poland; 3 Independent Researcher, Campinas 13023-210, SP, Brazil; caroldonadon.fono@gmail.com; 4 Department of Otolaryngology, Institute of Sensory Organs, 05-830 Warsaw, Poland; 5 Department of Psychobiology, Universidade Federal de São Paulo, São Paulo 04724-000, SP, Brazil; claudia.berlim@unifesp.br

**Keywords:** dyslexia, specific learning disorder, comorbidity, auditory-evoked potentials, hearing

## Abstract

**Background and Clinical Significance**: Developmental dyslexia is diagnosed through behavioral neuropsychological and language assessment, which depends on the child’s attention, motivation, and cooperation during testing. Auditory-evoked potentials provide objective, non-behavioral indices of neural encoding along the auditory pathway, but they are usually examined in group-level designs and in separate cohorts, so that intra-familial variability, and the level of the pathway at which atypical encoding emerges, remain poorly described. Characterizing siblings who share genetic background, family environment and schooling, using a single protocol that spans the brainstem, subcortical and cortical levels, is therefore clinically informative. **Case Presentation**: Two non-twin brothers (aged 13 and 11) with a clinical diagnosis of familial dyslexia and normal peripheral hearing underwent independent neuropsychological, speech–language and electrophysiological evaluations, comprising the click auditory brainstem response (ABR), the frequency-following response (FFR) to /da/ (80 dB SPL) in quiet and in ipsilateral and contralateral noise (masker 70 dB SPL; SNR ≈ +10 dB), and the P300 cognitive potential (auditory oddball). Click-ABR latencies and interpeak intervals were normal in both siblings, with an isolated, age-inappropriate V/I amplitude-ratio reduction in one. The FFR showed a condition-dependent, sibling-specific pattern of degradation, ipsilateral in one brother and contralateral in the other, with selective F0 collapse. The P300 was altered in both, with prolonged or absent components and low target-detection accuracy. All comparisons are descriptive. **Conclusions**: Preserved brainstem transmission contrasted with vulnerable subcortical speech encoding and altered cortical processing, revealing a central and biologically heterogeneous profile even within a family, and supporting the combined use of ABR, FFR and P300 as a complementary objective characterization of auditory processing in dyslexia.

## 1. Introduction

Reading involves a broad range of cognitive and linguistic processes related not only to decoding written stimuli but also to their comprehension. In its early stages, this process unfolds slowly and gradually, relying heavily on the phonological representation of written words. As these skills develop and consolidate, reading becomes increasingly automatized, allowing faster and more fluent word recognition. This progression enables the flow of information derived from reading to support the formation of new knowledge or the reorganization of previously established knowledge [1,2]. This wide range of abilities underlying accurate and effective reading highlights phonological processing as an essential component, grounded in phonological awareness, short-term and working phonological memory, and rapid lexical access. When impairments in phonological processing are identified, this clinical presentation is termed as dyslexia.

Specific Learning Disorder with impairment in reading, also referred to as dyslexia, is a neurodevelopmental disorder of neurobiological origin with a substantial hereditary component [3,4,5]. Its core features include deficits in the accuracy and fluency of word reading, which may also be accompanied by writing difficulties; these deficits are not attributable to sensory or intellectual impairment, nor to inadequate educational instruction [6,7]. The etiology of Specific Learning Disorder is considered multifactorial. Evidence indicates that its origin is not restricted solely to alterations in phonological processing but instead results from the interaction among several cognitive components [8,9]. Among these, impairments in broader neuropsychological functions, such as working memory, processing speed, and executive functions, may act together with genetic and environmental factors, contributing to the emergence and persistence of learning difficulties [10,11,12].

The diagnosis of developmental dyslexia currently relies on a comprehensive neuropsychological and speech–language evaluation, regarded as the gold-standard approach and typically encompassing measures of phonological awareness, phonological memory, rapid automatized naming, and reading/writing performance [8,9]. This behavioral approach, however, depends on the child’s active participation and can be influenced by attention, motivation, and cooperation during testing, which may limit its sensitivity in more complex clinical presentations. Auditory-evoked potentials provide objective, non-behavioral measures of neural encoding along the auditory pathway and have increasingly been proposed as a complementary tool alongside standard neuropsychological and language testing when characterizing the auditory and phonological processing profile of children with dyslexia [13,14].

Three specific gaps nevertheless remain. First, most electrophysiological studies in dyslexia are group-level comparisons between dyslexic and typical readers, so that the intra-individual and intra-familial variability of the electrophysiological phenotype is averaged and rarely described. Second, the successive stations of the auditory pathway are usually examined in separate cohorts and with different protocols; reports combining click-ABR, speech-evoked FFR and P300 in the same participants, in the same session and on the same equipment, remain scarce, although this is the design required to establish at which level of the pathway atypical encoding first emerges. Third, the FFR in dyslexia has been recorded almost exclusively in quiet or in ipsilateral noise; the contralateral-noise condition, which does not mask the signal at the test ear and therefore probes descending (efferent) modulation and binaural interaction rather than energetic masking, has seldom been applied to this population. Two siblings with familial dyslexia, assessed with an identical protocol, constitute an informative test case for these gaps, because genetic background, family environment, language input and schooling are largely shared, whereas the individual electrophysiological profile is free to vary.

This case report describes the neuropsychological, speech–language, and electrophysiological findings of two non-twin siblings both clinically diagnosed with developmental dyslexia through standard neuropsychological and language assessment. By examining two siblings who share a similar family and educational environment yet present with distinct ages and developmental profiles, this report aims to illustrate how audiological and electrophysiological assessment can complement the gold-standard behavioral battery in characterizing the auditory processing profile underlying their reading and language difficulties. Specifically, and in direct response to the gaps outlined above, the report describes, in the same two participants and within a single recording session, sensory transmission (click-ABR), subcortical speech encoding in quiet and under ipsilateral and contralateral noise (FFR), and cortical stimulus categorization (P300).

## 2. Detailed Case Presentation

This study was approved by the Institutional Research Ethics Committee (protocol code 7.115.963; approval date: 5 August 2025). The participants’ legal guardians were informed about the procedures to be performed during the assessments and agreed to their children’s participation by signing an Informed Consent Form (ICF). Data collection took place at the Language, Audiology, and Electrophysiology outpatient clinics of the Federal University of São Paulo (UNIFESP), in partnership with the Interdisciplinary Center for Child Neuropsychological Care (Núcleo de Atendimento Neuropsicológico Infantil Interdisciplinar, NANI).

Participants were two non-twin, monolingual Brazilian Portuguese-speaking brothers: Sibling 1, a 13-year-old boy attending the 6^th^ grade, and Sibling 2, an 11-year-old boy attending the 5^th^ grade. Both were referred for an evaluation because of marked and persistent difficulties in acquiring reading and writing skills. Their legal guardians reported a family history of learning difficulties on both the paternal and maternal sides. Both children attended public schools and received standard educational instruction.

Both siblings underwent comprehensive, independent neuropsychological, speech–language, and electrophysiological evaluations. A battery of standardized instruments, normed and validated for the Brazilian population, was administered to assess distinct cognitive and linguistic domains. The neuropsychological evaluation focused on general intellectual functioning, attention, executive functions, memory, and visuoconstructive skills.

## 3. Clinical Assessment

### 3.1. Neuropsychological Evaluation

General intellectual functioning was assessed in Sibling 1 using the Wechsler Abbreviated Scale of Intelligence (WASI) [15] and in Sibling 2 using the Wechsler Intelligence Scale for Children—Fourth Edition (WISC-IV) [16]. Attention was assessed in both siblings using a computerized Continuous Performance Test (CPT), and executive functions were assessed using the Behavior Rating Inventory of Executive Function (BRIEF) [17]. Verbal learning and memory were assessed using the Rey Auditory Verbal Learning Test (RAVLT) [18], and visuospatial memory was assessed through delayed recall on the Rey Complex Figure Test [19]. Digit-span and Corsi block-tapping tasks were used to assess working memory [20]. Visuoconstructive skills, along with behavioral and adaptive functioning, were assessed using the Rey Complex Figure copy trial [19], the Swanson, Nolan and Pelham Rating Scale—IV (SNAP-IV) [21], and the Child Behavior Checklist [22], respectively.

Different Wechsler instruments were used for the two siblings because the neuropsychological evaluations were conducted independently, within routine clinical care, and the instrument was selected according to the referral question of each child. For Sibling 1, whose referral was restricted to persistent reading and writing difficulties, with no indication of global developmental delay in his school history, the abbreviated scale (WASI) was considered sufficient to estimate general intellectual functioning and to verify that reading performance was discrepant from overall cognitive level, as required for a diagnosis of Specific Learning Disorder. For Sibling 2, whose referral described global academic failure and who remained illiterate, the complete battery (WISC-IV) was necessary in order to obtain the four index scores (Verbal Comprehension, Perceptual Reasoning, Working Memory and Processing Speed), to rule out intellectual disability and to characterize working memory and processing speed in detail. Both instruments are normed and validated for the Brazilian population, share the same theoretical model and metric (M = 100, SD = 15), and the abbreviated scale provides a Full-Scale IQ estimate that correlates strongly with the corresponding complete scale. Because the two instruments nevertheless differ in composition and in the number of subtests, the FSIQ values reported here are interpreted only against each participant’s own age norms, for classification purposes, and are not compared directly with one another.

Sibling 1’s general cognitive profile was classified as average (Full-Scale IQ [FSIQ] = 95), revealing a discrepancy relative to his academic performance. His profile showed considerable complexity, with clinically evident difficulties in attention and executive functions, particularly cognitive flexibility and everyday working memory.

Sibling 2’s evaluation revealed a more severe and globally impaired neurodevelopmental profile. His assessment showed borderline intellectual functioning (FSIQ = 77), with severe working-memory deficits, marked inattention, and executive dysfunction.

### 3.2. Speech and Language Evaluation

Speech and language assessment were carried out through a battery of oral language tests. Expressive vocabulary was assessed using the Children’s Naming Test [23], while receptive vocabulary was measured using the Picture Vocabulary Test [24]. Narrative discourse was assessed through a story-generation task, and speech-sound production was assessed using the ABFW Child Language Test Battery [25].

Phonological processing was assessed through several tests. Phonological awareness was evaluated using the Oral Production Phonological Awareness Test [23], which measures syllabic- and phonemic-level skills. Short-term phonological memory was assessed using the Children’s Phonological Working Memory Test [26]. Lexical access was assessed using the Rapid Automatized Naming Test (RAN) [27], which measures naming speed for colors, objects, letters, and numbers.

Word reading was assessed using the Word and Pseudoword Decoding Assessment Protocol [28,29], and writing was assessed using the dictation-writing subtest of the School Performance Test—Second Edition (TDE-II) [30]. Finally, syntactic awareness was assessed in both siblings using the Syntactic Awareness Test [23], aimed at evaluating grammatical-judgment skills.

Regarding oral language, Sibling 1 performed within average limits, including vocabulary; however, deficits characteristic of a dyslexia diagnosis were observed, namely severe impairment in phonemic awareness and markedly slow Rapid Automatized Naming (RAN) for alphanumeric stimuli. Taken together, these findings indicate a classic dyslexia profile for Sibling 1, characterized by severe and specific impairment in word and pseudoword reading and writing, alongside strong, above-average mathematical skills.

Sibling 2 presented profound impairment in expressive vocabulary, whereas receptive skills remained within average limits. Phonological processing showed severe deficits across all measures of awareness, memory, and RAN. This combination of findings culminated in substantial academic impairment: Sibling 2 remained illiterate, at a pre-alphabetic stage, with correspondingly severe deficits in mathematics.

## 4. Electrophysiological Evaluation

The audiological and electrophysiological test battery was performed at the Audiology and Electrophysiology Clinic of the Escola Paulista de Medicina, UNIFESP, São Paulo, Brazil. Hearing thresholds were within normal limits (≤20 dB HL from 250 to 8000 Hz) as measured with a Pello™ audiometer (GSI Grason-Stadler, Eden Prairie, MN, USA), and word-recognition scores were within normal limits [31]. Tympanometry revealed type-A tympanograms [32], and ipsilateral and contralateral acoustic reflexes were present bilaterally [32], obtained using a Tympstar Pro™ (GSI Grason-Stadler, Eden Prairie, MN, USA) immittance meter.

Electrophysiological assessments were conducted in an acoustically and electrically shielded room, with participants comfortably seated in a reclining chair. The procedures included recording the auditory brainstem response (ABR) to click stimuli and the long-latency auditory-evoked potential (P300) using the Neuro-Audio™ System (Neurosoft, Ivanovo, Russia), as well as the frequency-following response (FFR) using the Bio-logic™ Navigator Pro (Natus Medical, Middleton, WI, USA). Electrode placement followed the International 10–20 System [33], with the montage varying according to the specific protocol of each test. During recordings, electrode impedance was maintained below 5 kΩ, and interelectrode impedance difference remained below 3 kΩ. Each assessment protocol and its results will be detailed in the following sections. The parameters used for the respective protocols are presented in Table 1.

Because the present battery samples successive levels of the auditory pathway, it is useful to situate its components within the conventional latency-based classification of auditory-evoked potentials before the protocols are described, since two classes of that classification were not recorded here. Short-latency potentials (0–10 ms) are represented in this report by the click-ABR, whose successive waves index sensory transmission and neural synchrony from the auditory nerve to the rostral brainstem. Middle-latency potentials (auditory middle-latency response, AMLR; approximately 10–50 ms; components Na, Pa and Nb) index the thalamo-cortical projection and the primary auditory cortex, whereas exogenous long-latency potentials (auditory late response, ALR; approximately 50–250 ms; complex P1–N1–P2) index the arrival and the passive detection of the acoustic event at the auditory cortex; normative latency and amplitude values for the short-, middle- and long-latency classes recorded under comparable conditions have been reported previously [14]. The P300 recorded here belongs to the long-latency family in terms of its time window, but is endogenous rather than exogenous: it is not obligatorily evoked by the acoustic event and is generated only when the listener detects and categorizes an infrequent, task-relevant stimulus, so that it indexes attentional allocation and stimulus evaluation rather than the arrival of sound at the cortex [34,35]. The FFR does not belong to this sequence of transient potentials at all: it is a sustained response that phase-locks to the periodicity of the stimulus throughout its duration, is generated predominantly in the rostral brainstem with a documented cortical contribution [36], and is therefore the only measure in the battery that reports how the spectral and temporal detail of speech is encoded, rather than whether a sound has been transmitted or detected [37]. The AMLR and the exogenous ALR were not recorded in the present protocol.

A general remark on the interpretation of the numerical values reported below is required. This is a report of two cases; no inferential statistics were applied, and none of the comparisons presented between siblings, between ears or between listening conditions were tested for statistical significance. All numerical differences, percentage changes and correlation coefficients are therefore descriptive observations derived from single recordings, reported to characterize each participant’s profile against the normative references cited, and not to establish that a difference is reliable or biologically significant.

### Click Auditory Brainstem Response (Click-ABR)

**Figure 1:**
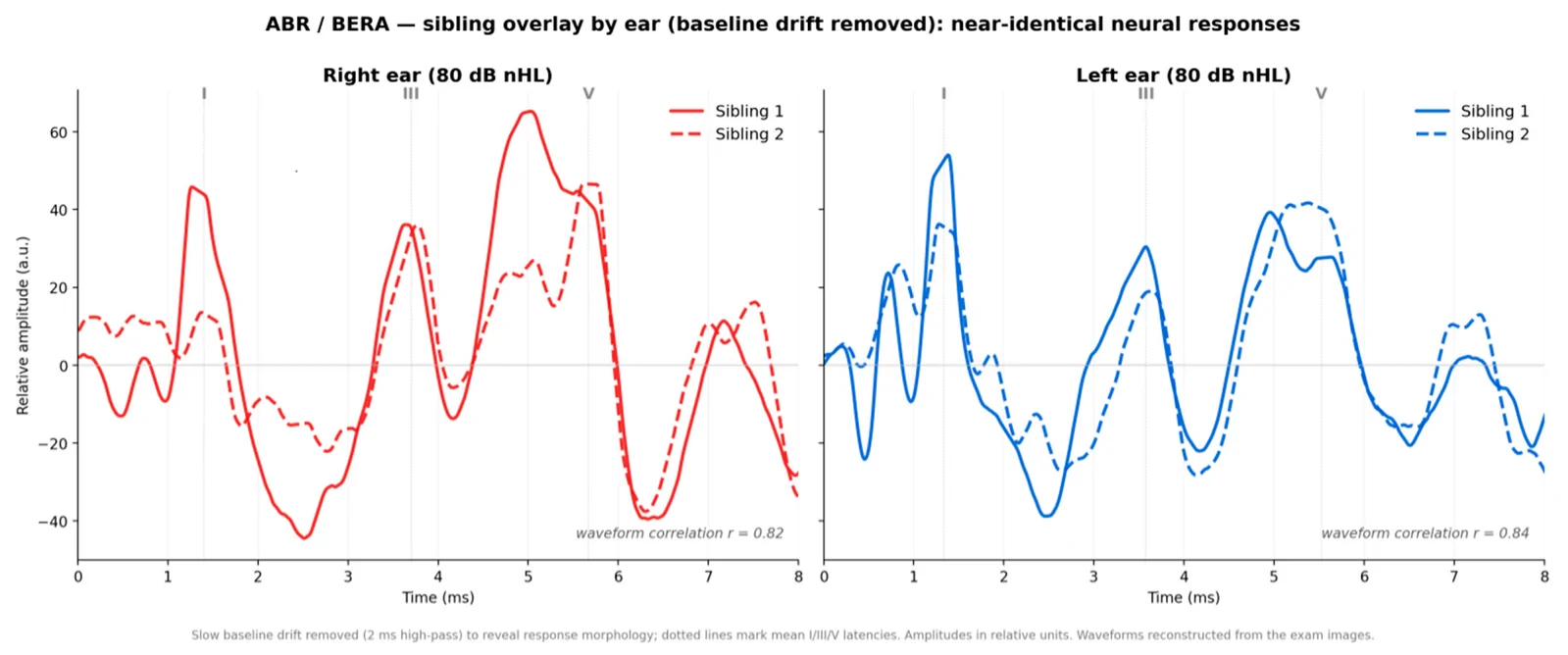
Click-ABR waveforms of the two siblings in both ears, with waves I, III and V labeled.

ABR assessment revealed results within normal limits in both siblings, considering the latency and amplitude values of waves I, III, and V, as well as the I–III, III–V, and I–V interpeak intervals, in both ears [38]. Waveform overlays for each ear, illustrating the near-identical morphology between siblings (right ear: r = 0.82; left ear: r = 0.84), are presented in Figure 1. The interaural wave V latency difference also remained within normal limits in both siblings, consistent with the other findings. However, distinct results were observed regarding the V/I amplitude ratio in Sibling 2’s left ear, with a value below the established normative parameters [39]. This finding was not observed in the right ear, nor was it observed bilaterally in Sibling 1. The ABR results are presented in Table 2.

## 5. Frequency Following Response (FFR)

**Figure 2:**
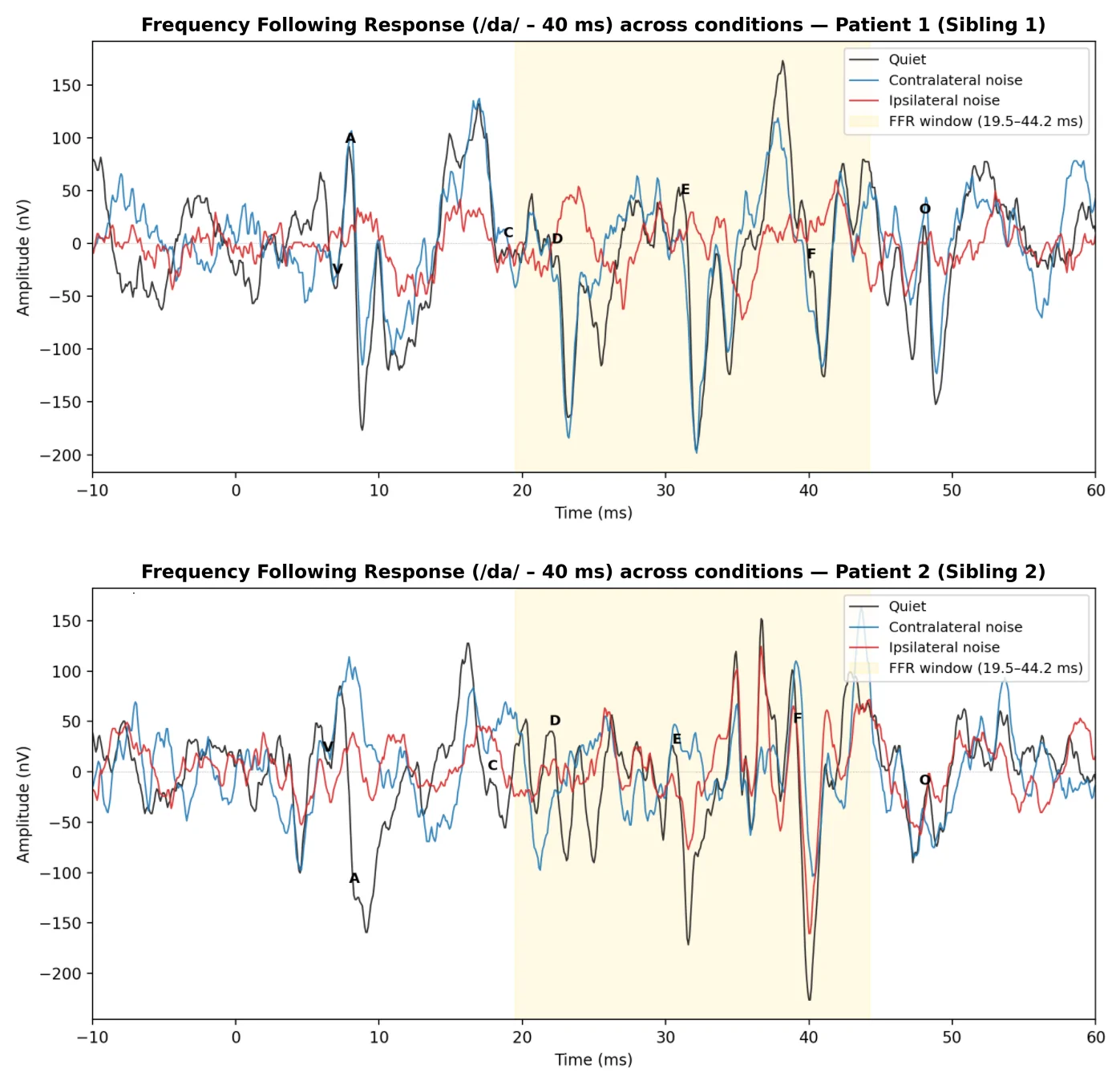
Speech-evoked FFR to /da/ across the three listening conditions (quiet, ipsilateral noise, contralateral noise): (**upper**) Sibling 1 and (**lower**) Sibling 2. Components V–O are labeled. The individual markers indicate the seven components of the response: V and A form the onset complex evoked by consonant release (V = positive peak; A = the following trough); C marks the transition between the consonant burst and the vowel; D, E and F are the peaks of the sustained, frequency-following portion, which track the periodicity of the vowel and are separated by approximately one period of the fundamental frequency; and O is the offset response, occurring after stimulus termination. Latency and amplitude were measured at each marker; markers are labeled separately for each listening condition, and a rightward displacement of a marker across conditions represents a delay of the corresponding component.

FFR recordings were obtained under three conditions: (i) quiet, (ii) contralateral white noise, and (iii) ipsilateral white noise (Figure 2). The /da/ stimulus was presented at 80 dB SPL, and the masking noise at 70 dB SPL in both noise conditions (SNR ≈ +10 dB), such that the ipsilateral and contralateral conditions were matched for masker level and differed only in the route of noise presentation. Each analyzed waveform was derived from the sum of two replicated averages of 3000 artifact-free sweeps each. Identification of the seven response components (V, A, C, D, E, F, and O) was performed on the 6000-sweep grand-average waveform; raters conducted visual inspection and manual marking of peak latencies and amplitudes. Data were subsequently processed using AEPASC II software (version 1.6.0) and exported as numerical values in txt format. All further data processing was carried out in MATLAB (version R2017b) and Python (version 3.6) using the Brainstem Toolbox, which allowed additional analyses, including the RMS amplitude and signal-to-noise ratio (SNR) of the sustained response region, spectral amplitudes within the F0, F1, and higher-harmonic bands, and cross-correlation (correlogram) analysis between responses obtained in quiet versus noise conditions.

Because the auditory brainstem fires in synchrony with the periodicity of a complex sound, the scalp-recorded response reproduces the acoustic structure of the eliciting syllable, so that the recorded waveform physically resembles the stimulus. Two portions are distinguished. The onset portion (components V and A, at approximately 6–10 ms) is the synchronous response to the abrupt acoustic change at consonant release and is read in the same way as wave V of the click-ABR: later or smaller responses indicate less precise neural synchrony, while the interval and the slope between V and A quantify how sharply this transition is encoded. The sustained portion (components C, D, E and F, followed by the offset component O) reflects phase-locking to the periodic vowel; consecutive peaks in this region are separated by approximately one period of the speaker’s voice pitch, so this portion carries the fundamental frequency (F0, perceived as voice pitch and prosody) and the first formant (F1, which conveys vowel identity). In practical terms, a prolonged V–A complex with a flatter slope indicates imprecise timing of the consonant transition, whereas a reduction in F0 or F1 magnitude indicates an impoverished neural representation of the periodic and formant information of the vowel.

The rationale for recording the FFR under two different routes of masking is presented here, before the results. Ipsilateral noise is delivered to the same ear as the speech stimulus and therefore produces energetic masking at the cochlea and along the ascending pathway of the test ear: it degrades the signal itself and tests how robust subcortical encoding remains when the acoustic input is impoverished, which is the situation of a child listening to a teacher in a noisy classroom. Contralateral noise, by contrast, is delivered to the opposite ear and does not mask the stimulus at the test ear; any change it produces in the response must therefore be mediated by binaural interaction and by descending modulation, in particular the medial olivocochlear efferent reflex, which adjusts cochlear gain in the stimulated ear in response to sound in the opposite ear and is one of the mechanisms proposed to improve the signal-to-noise ratio during listening to noise. Recording both conditions at the same masker level (70 dB SPL; SNR ≈ +10 dB), so that they differ only in route of presentation, allows two potentially dissociable sources of vulnerability to be separated: degradation of encoding under direct acoustic interference (ipsilateral) versus inefficiency of descending and binaural modulation (contralateral). This dissociation was the reason for including both conditions and is the basis on which the sibling-specific patterns described below are interpreted.

Peak identification followed a standardized procedure. Components V, A, C, D, E, F and O were marked by visual inspection of the 6000-sweep grand-average waveform. Two experienced audiologists, both trained in speech-evoked potentials, marked every waveform independently and without access to each other’s markings. Marking was performed blind: neither the identity of the participant nor the listening condition to which each waveform corresponded was disclosed to the raters beforehand. The two sets of marks were then compared component by component: where the raters converged, the marking was retained; whenever they diverged, the recording was submitted to a third and more experienced rater, the professor supervising the electrophysiology service, who re-inspected the response and issued the final decision, which was the value adopted. The same independent-marking and adjudication procedure was applied to the click-ABR (waves I, III and V) and to the P300 (P3a and P3b). Agreement between the two independent raters was not quantified by a reliability index, since the procedure was designed to resolve divergence through adjudication rather than to estimate reliability.

Waveform similarity was quantified as follows. Before correlation, each averaged waveform was band-pass filtered over the same range used during acquisition (100–2000 Hz) and baseline-corrected by subtraction of the mean of the pre-stimulus interval, in order to remove any direct-current offset; the two waveforms entering each comparison were placed on a common time base, with an identical sampling rate and identical analysis window. No amplitude normalization was applied, so that the coefficients reflect the covariation in the raw averaged waveforms, and no artifact rejection was applied beyond the rejection performed online during acquisition. Similarity was then computed as the Pearson product-moment correlation coefficient between the two waveforms, point by point across the analysis epoch, at zero lag: no time-shift correction was applied, so that a temporal displacement between two responses is preserved in the comparison rather than compensated for. The coefficient is therefore sensitive both to differences in morphology and to differences in timing, which is the reason why negative values occur. In a response that is quasi-periodic over its sustained portion, a latency shift corresponding to a fraction of the period of the fundamental frequency is sufficient to place the two waveforms in opposite phases; a negative coefficient consequently indicates phase misalignment of a response that is present, and not an inverted or absent response. Finally, two conceptually different families of comparison are reported and are kept distinct throughout: within-sibling comparisons, in which the response of one participant in quiet is compared with the response of the same participant in noise, and between-sibling comparisons, in which the responses of the two participants are compared within the same listening condition.

Responses under the three assessment conditions:

(i) Quiet* *condition: Under the ideal listening condition, both siblings exhibited identifiable seven components (V–O), with a robust fundamental frequency; however, the waveform correlation between siblings was considered moderate (r = 0.37).
(ii) Contralateral noise condition: Stimulation in the presence of contralateral white noise revealed a differentiated response between siblings. The comparison between Sibling 1’s responses in the quiet versus contralateral noise conditions showed high similarity (r = 0.89), whereas for Sibling 2, the presence of contralateral noise markedly altered the responses (r = 0.42).
(iii) Ipsilateral noise condition: Stimulation in the presence of ipsilateral white noise reversed the response pattern between siblings. Sibling 1 exhibited a degradation of responses, with a delay of approximately 2 to 4 milliseconds across all components and a low correlation with the responses obtained in quiet (r = −0.08), whereas for Sibling 2, both waveform morphology and response timing were preserved under the ipsilateral noise condition (r = 0.78).

The combined analysis of results revealed that the moderate similarity in responses between siblings (r = 0.37) was further compromised by the introduction of noise, decreasing under contralateral noise (r = 0.06) and under ipsilateral noise (r = −0.15). Regarding the quantitative FFR results, the following values were derived from the 6000-sweep summed waveform (latencies and amplitudes marked in AEPASC II), condition by condition, and summarized in Table 3. In quiet, both siblings exhibited identifiable seven components (V–O), with a robust F0 (Sibling 1: 74 nV; Sibling 2: 51 nV) and a well-defined V–A slope (Sibling 1: −0.30 µV/ms; Sibling 2: −0.14 µV/ms). In Sibling 1, degradation was specific to the ipsilateral condition, characterized by prolongation of wave V (7.12→8.53 ms) and wave A (8.03→10.62 ms), widening of the V–A interval (0.91→2.09 ms), attenuation of the V–A slope (−0.30→−0.04 µV/ms), and a 69% decline in F0 (74→23 nV), whereas the contralateral condition preserved latencies, slope (−0.27), and F0 (72 nV).

**Figure 3:**
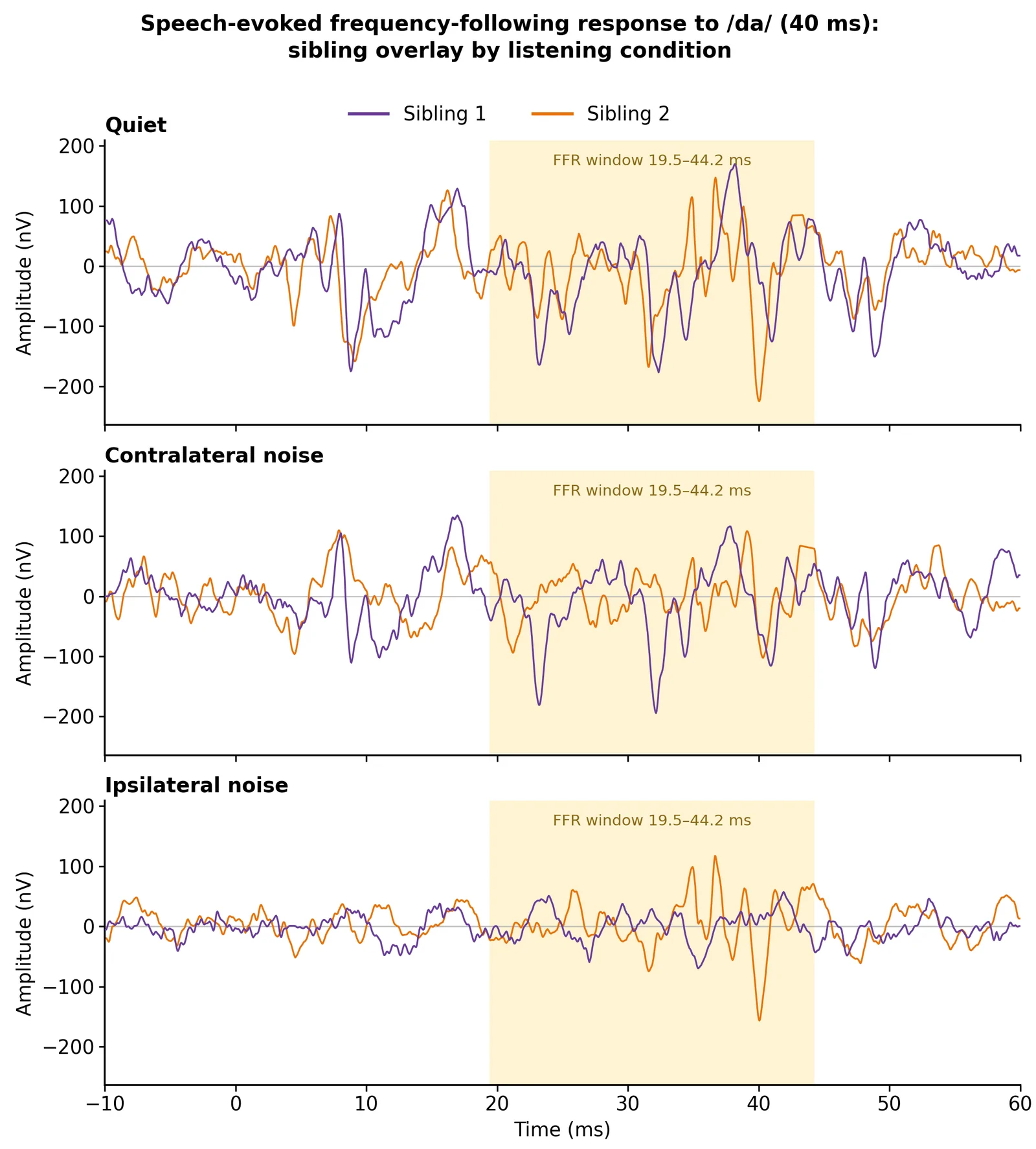
Speech-evoked frequency-following response (FFR) to the 40 ms /da/ syllable: sibling overlay by listening condition. Waveforms of Sibling 1 (purple) and Sibling 2 (orange) are superimposed in quiet (**top**), contralateral noise (**middle**) and ipsilateral noise (**bottom**). The shaded band (19.5–44.2 ms) marks the sustained (frequency-following) analysis window.

In Sibling 2, the most pronounced alteration occurred under the contralateral condition, particularly in the spectral domain: F0 decreased by 78% (51→11 nV), far exceeding the reduction observed under ipsilateral noise (−31%), accompanied by prolongation of wave V (6.45→7.53) and wave A (8.28→9.53) and near-abolition of wave A (amplitude −0.02 µV, versus −0.16 in quiet). The V–A slope, however, remained attenuated under both noise conditions (ipsilateral: −0.05; contralateral: −0.06 µV/ms), indicating that Sibling 2’s contralateral signature is primarily spectral and latency-based, rather than reflecting a change in the slope of the V–A complex. Figure 3 displays the FFR waveform overlays for both siblings across the three conditions (quiet, contralateral noise, and ipsilateral noise).

### Cognitive Evoked Potential (P300)

P300 responses were obtained using the auditory oddball paradigm, in which two stimuli were presented in pseudorandom sequence: a frequent, non-target stimulus and a rare, target stimulus, occurring in an 80/20 ratio, respectively. Siblings were instructed to respond only upon detecting the target stimulus. Reaction time corresponds to the interval between stimulus presentation and the moment the patient presses the response button upon identifying the target stimulus and is widely used as a behavioral correlate of the P3b component. Reaction time variability was further quantified using the root-mean-square (RMS) deviation of reaction times across trials, providing an index of response consistency independent of mean latency or accuracy.

P300 is not a response to the sound as such, but to the act of recognizing it: it is elicited only when the listener detects an infrequent, task-relevant stimulus among frequent ones, and its latency indexes the time required to categorize that stimulus, independently of the motor response. The reaction time recorded here includes both this categorization time and motor execution, which is why the two measures are related but not interchangeable. The P3a subcomponent, earlier and frontally distributed, is associated with automatic orienting of attention towards the deviant stimulus, whereas the P3b subcomponent, later and temporo-parietally distributed, is associated with conscious evaluation of the stimulus and updating of working memory. P3a and P3b were marked by the same two raters and following the same consensus procedure described above for the FFR, and the values reported below are descriptive.

**Figure 4:**
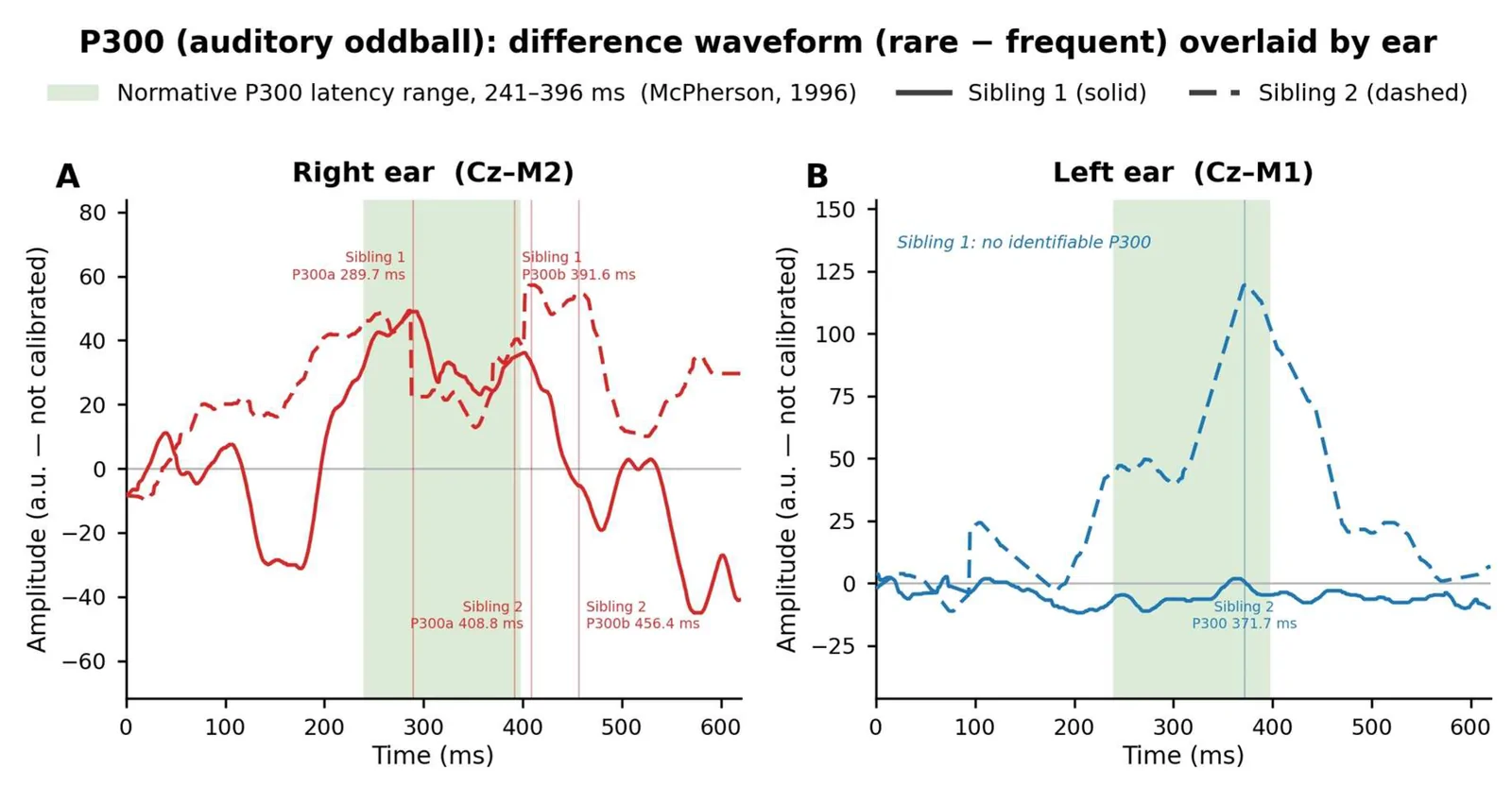
P300 (auditory oddball) event-related waveforms—rare, frequent and difference traces, with P300a/P300b marked: (**A**) Sibling 1 and (**B**) Sibling 2.

P300 assessment revealed alterations in the response pattern in both siblings. Both the P300a and P300b waves were identified in the results obtained. Regarding latency, Sibling 1 showed P300a and P300b values of 289.7 ms and 391.6 ms in the right ear, respectively, within normal limits for age [34], while the P300 wave was absent in the left ear (Figure 4). Sibling 2, in turn, showed increased P300a and P300b latencies in the right ear (408.8 ms and 456.4 ms, respectively), exceeding the normative values expected for age [36], whereas the left ear showed a P300 wave within normal limits, recorded at 371.7 ms (Figure 4).

Regarding reaction time and response consistency (Table 4), Sibling 1 showed a mean reaction time to significant stimuli in both ears (367 ms in the right ear and 399 ms in the left ear); however, response consistency was poor bilaterally, reflected in a low percentage of correctly identified significant stimuli (46.6% in the right ear and 43.4% in the left ear) and an elevated RMS deviation (103 ms in the right ear and 90.3 ms in the left ear). Sibling 2 showed a similar mean reaction time to significant stimuli for both ears (356 ms in the right ear and 355 ms in the left ear), but response consistency also remained poor, with 38.8% correctly identified significant stimuli in the right ear and 53.7% in the left ear, and RMS deviations of 117 ms in the right ear and 88.9 ms in the left ear.

Difference traces (rare-frequent) extracted from the exam images; the time axis was calibrated in ms from the labelled P300 components. Read for LATENCY: amplitude is not calibrated, because the P300 display auto-scales each trace, so peak heights are not comparable between traces or between ears. Colour marks the ear (red-right, blue-left) and line style marks the sibling (solid Sibling 1, dashed = Sibling 2). The shaded band is the normative P300 latency range of McPherson (1996) [34], 241–396 ms; the vertical markers give the component latencies reported in Table 4.

**Figure 5:**
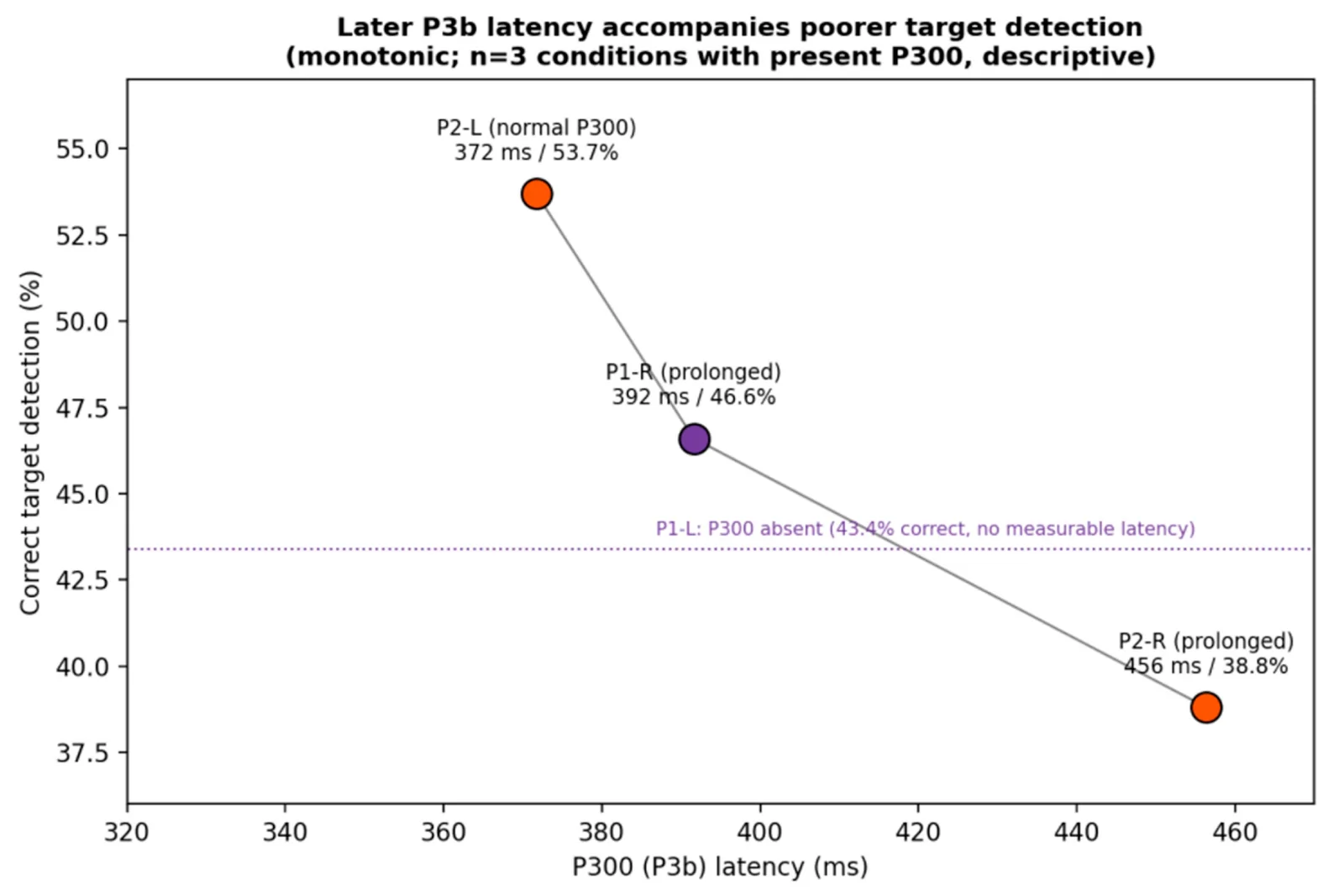
Relationship between P3b latency and target-detection accuracy across ear conditions (descriptive). Legend: ms= milliseconds; L = left ear; R = right ear.

Latency classification considered the age of each participant. Sibling 2 (11 years old) is fully covered by the 5–12-year normative range (241–396 ms): his right ear showed a P300b latency of 456.4 ms, exceeding the pediatric ceiling by 60.4 ms and indicating unequivocal prolongation, whereas the left ear (371.7 ms) remained within the pediatric limit (24.3 ms below the ceiling) and was classified as normal (Figure 5). Sibling 1 (13 years old) falls within an age range for which McPherson (1996) [34] does not provide a specific norm; for this participant, a dual comparison against the adjacent normative ranges (5–12 years: 241–396 ms; 17–30 years: 225–365 ms) was adopted. His only identifiable component (right ear, P300b: 391.6 ms) remained within the pediatric norm (≤396 ms, only 4.4 ms below the ceiling), although it exceeded the adult limit (365 ms) by 26.6 ms; no identifiable component was obtained in the left ear (absent response). The pediatric reference was chosen based on two converging considerations: age proximity—13 years is only one year from the upper limit of the pediatric range and four years from the lower limit of the adult range (17–30 years)—and the maturational trajectory of P300 latency, which decreases continuously but at a decelerating rate throughout adolescence, reaching adult values only in early adulthood [40]. Applying the adult limit (365 ms) to a 13-year-old adolescent still undergoing maturation would risk a false-positive classification of latency prolongation.

**Figure 6:**
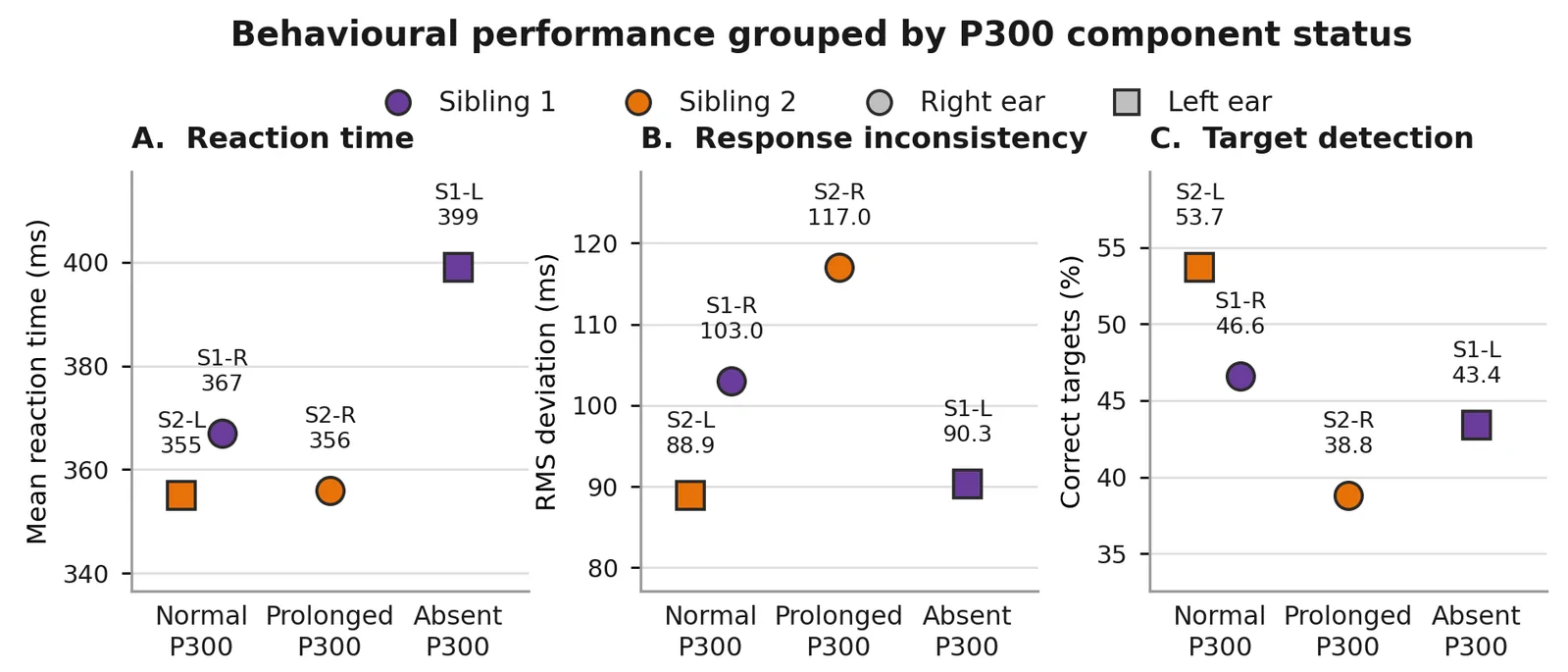
P300 behavioral performance by component status (normal/prolonged/absent): (**A**) reaction time, (**B**) RMS deviation, (**C**) target-detection accuracy. Sibling 1 (purple) and Sibling 2 (orange); circles = right ear, squares = left ear. Descriptive (*n* = 1 per cell). Legend: ms = milliseconds; L = left; R = right.

Behavioral performance consistently mirrored this latency classification in terms of accuracy. The two conditions with normal latency accounted for the highest hit rates in the sample: Sibling 2’s left ear (53.7%) and Sibling 1’s right ear (46.6%), which were both higher than the altered conditions (absent P300: 43.4%; prolonged: 38.8%) (Figure 6). The parallel between Sibling 2’s left ear—reclassified as normal based on the pediatric reference, and Sibling 1’s right ear, normal according to the directly applicable norm, suggests that normal P300b latency coincides with higher behavioral accuracy even across participants of different ages and ears.

The condition with unequivocal prolongation (Sibling 2, right ear) displayed the opposite, internally coherent profile of processing degradation: lower accuracy (38.8%) and greater variability (RMS of 117 ms), despite a short reaction time (356 ms), a pattern consistent with a hasty, less controlled response. It should be noted, however, that the variability in Sibling 1’s right ear (RMS of 103 ms) did not parallel its good accuracy, indicating that reaction time, RMS deviation, and hit rate are not redundant with one another, nor with latency. This lack of redundancy, together with the coherence observed in accuracy, provides preliminary justification for standardizing these behavioral measures as complementary, rather than merely accessory, parameters to P300 latency in the assessment of central auditory processing.

However, it is relevant to note that there is no consolidated clinical norm for reaction time, hit rate, or RMS deviation in the P300 paradigm; the only measure with an age-dependent norm is component latency [34]. As previously described, reaction time reflects both stimulus categorization and motor execution; for this reason, P300b latency, which precisely indexes the stimulus categorization and evaluation process, tends to parallel reaction time in this type of task [35,41]. Given the absence of norms for these parameters, a relational approach was adopted: conditions were grouped according to the patient’s own P300 response (normal, prolonged, or absent), and behavioral performance was then compared across these groups.

Each point is one ear-condition: S1 and S2 denote Sibling 1 and Sibling 2, circles the right ear and squares the left ear Component status was classified against the paediatric norm of McPherson (1996) [34] (5–12 years, 241–396 ms), applied to both siblings; Sibling 1, aged 13 years, was assigned to the nearest and most conservative norm. Both conditions with a normal 300 hold the two highest accuracies, whereas the prolonged condition (S2-R) shows the highest RMS deviation and the lowest accuracy despite a short reaction time. Descriptive observations only (n = 1 per cell).

## 6. Discussion

Developmental dyslexia has a substantial genetic component, extensively documented by family and twin studies, with heritability estimates generally ranging between approximately 40% and 70%, depending on the methodology and sample characteristics considered [42,43,44]. Classic twin studies, such as those conducted in the Colorado Twin Study by DeFries and Alarcón (1996) [45], demonstrate substantially higher concordance rates between monozygotic than dizygotic twins, providing robust evidence for a genetic contribution to specific reading difficulties. Consistently, genome-wide association studies (GWAS) have identified multiple loci associated with dyslexia, reinforcing its polygenic architecture and including, among the historically described candidate genes, DYX1C1, KIAA0319, DCDC2, and ROBO1 [42,44,46]. Furthermore, family history stands out as one of the most consistent risk factors for the disorder: children with a father, mother, or sibling with dyslexia have a significantly higher probability of developing reading difficulties, with studies indicating prevalences of around 40–60% in this population, in contrast to considerably lower rates observed in children without a family history, within prevalence ranges typical of the general population [47,48,49].

The multifactorial nature of dyslexia is a widely discussed topic, and current theories indicate that, beyond the core phonological deficit, growing evidence suggests that dyslexia involves alterations in auditory processing at multiple levels of the pathway, from sensory encoding to the cognitive processing of sound information [10,50]. Auditory-evoked potentials provide objective and complementary indices of these levels: the click-evoked auditory brainstem response (ABR) assesses the integrity of sensory transmission and neural synchrony in the more peripheral portions of the pathway [39,51,52]; the Frequency-Following Response (FFR) reflects, with high fidelity, the subcortical encoding with a cortical contribution as discussed by Coffey et al. [36] of the temporal and spectral characteristics of speech (the envelope/fundamental frequency [F0], responsible for pitch and prosodic information, and the spectral energy in the formant region, particularly the rapid transition of the second formant [F2], which enables analysis of place-of-articulation cues), and can be assessed under ideal or degraded listening conditions (presence of noise) [36,37,53,54,55]; and the long-latency cognitive potential (P300) indexes cortical processes of attention, discrimination, and stimulus categorization [34,35,36,40]. Together, these procedures allow an analysis of auditory stimulus perception across the auditory pathway. Given this association between dyslexia and family history, the present study aimed to analyze the auditory and electrophysiological findings of two siblings diagnosed with dyslexia.

### 6.1. Electrophysiological Assessment of Hearing

Combined electrophysiological assessment makes it possible to localize the level of the auditory pathway at which impairment may exist; in the present study, this analysis allowed the auditory pathway to be examined from the brainstem to the cortex. Analysis of the siblings’ evoked potentials showed that the peripheral portion was preserved, whereas higher-order regions, namely subcortical and cortical encoding, were found to be altered.

### 6.2. Click Auditory Brainstem Response (Click-ABR)

Click-ABR showed absolute latencies and interpeak intervals (I–III, III–V, I–V) within normal limits in both siblings and in both ears, confirming the integrity of sensory transmission and neural synchrony in the peripheral and brainstem portions of the auditory pathway. This finding is expected and consistent with the literature, since, in children with dyslexia and reading difficulties, the brainstem response to simple transient stimuli (clicks) is typically normal, whereas alterations emerge in the response to complex speech sounds, particularly under demanding conditions (noise)[54, 56,57]. The normal click-ABR therefore supports the interpretation that the impairment observed in this report is central in nature and dependent on stimulus complexity and context, rather than reflecting a low-level acuity or sensory-conduction deficit.

The only click-ABR parameter outside the reference standard was the V/I amplitude ratio in Sibling 2’s left ear (0.68; below the reference value of ≥1 proposed by Hall, 2015 [39]). Wave I reflects the activity of the distal auditory nerve, and wave V reflects the activity of the rostral portion of the brainstem (lateral lemniscus–inferior colliculus) [52]; the V/I ratio thus expresses the relationship between peripheral input and rostral brainstem output. According to Hall (2015) [39], wave I may exceed wave V up to approximately 2 years of age, the period during which ABR maturation is completed; from that point on, with the ABR fully matured, a V/I ratio ≥1 is expected. Therefore, the presence of a V/I ratio <1.0 in an 11-year-old individual, as observed in Sibling 2, departs from the pattern expected for age and should be regarded as a warning sign.

This age-inappropriate finding warrants that it be recorded and monitored rather than simply dismissed. In this sense, this age-inappropriate reduction in the V/I ratio may represent an early indication, already at the brainstem level, of involvement of higher auditory stations; such involvement is indeed evidenced, within this same report, by the subsequent FFR and P300 findings, which document alterations in subcortical encoding and cortical processing of auditory information. This limited sensitivity of the isolated click-ABR is consistent with what has been reported in a recent systematic review of auditory electrophysiology in Developmental Language Disorder, in which conventional click-ABR also failed to differentiate groups in most studies, and was recommended more as a peripheral-pathway screening procedure than as an isolated differential marker [58].

### 6.3. Frequency Following Response

In the present report, FFR assessment revealed a pattern of subcortical speech encoding that was strongly dependent on the listening condition and differentiated between siblings, despite the shared dyslexia diagnosis and preserved peripheral audiological profile. Under the quiet condition, both siblings showed typical morphology, with the seven components (V–O) clearly identifiable and a robust fundamental frequency, suggesting that, in the absence of signal degradation, the basic integrity of phase-locking in the auditory brainstem was preserved in both. These results converge with evidence that children with dyslexia may show relatively preserved encoding of voice pitch and V–O morphology under ideal listening conditions, but with more evident impairment when the stimulus is degraded or when the context demands greater use of figure–ground temporal cues [53]. This finding is also reflected in the reduced F1 values observed in both siblings under the noise condition that was most unfavorable for each of them (Table 3), consistent with a vulnerability in the neural representation of formant information under increased auditory demand, as occurs, for example, in the classroom [54].

When stimulation with speech stimuli occurred in the presence of noise, a differentiated pattern was observed in the siblings’ responses. In Sibling 1, contralateral noise did not substantially alter the response to the speech stimulus; however, when presentation was ipsilateral, there was a degradation of the response in the sustained portion of the FFR, which is related to the periodic pattern of speech (fundamental frequency and harmonics); when altered, this would be related to impaired performance in speech-in-noise perception and in the reading process [59].

In this configuration, noise disorganizes neural synchrony along the ipsilateral pathway, generating timing delays and reduced stability in the subcortical encoding of the first formant and harmonics—elements that are critical for pitch perception, prosody, and speech segmentation—and may therefore contribute to difficulties in speech-in-noise processing and in reading in children with dyslexia [60].

The magnitude of this desynchronization can be appreciated by relating two measures that were obtained independently of one another. The sustained portion of the FFR follows the fundamental frequency of the stimulus, so that consecutive peaks are separated by approximately one period, of the order of 9 ms for the /da/ syllable used here. A displacement of 2 to 4 ms, as observed in Sibling 1 under ipsilateral noise, therefore corresponds to a substantial fraction of one period and is by itself sufficient to bring the response recorded in noise out of phase with the response recorded in quiet. The near-zero coefficient obtained for this comparison (r = −0.08) is consistent with that displacement, so that the latency measurements and the waveform correlation describe the same phenomenon by two independent routes: not an abolition of the response, but a loss of the temporal precision with which the periodicity of the vowel is encoded.

Sibling 2, in turn, showed the opposite pattern, with relatively stable subcortical speech encoding in the presence of ipsilateral noise, but degraded responses under contralateral noise, which would be related to a difficulty in binaural integration associated with the need to suppress noise input in the opposite ear (descending modulation) [61]. This specific vulnerability in the binaural configuration suggests a difficulty in integration between the two ears and in the efficient engagement of descending modulation mechanisms (rostral efferent system), which have been identified, in studies using FFR in contralateral noise, as crucial for improving the signal-to-noise ratio in speech-in-noise and dichotic listening situations. The literature data on FFR in noise show that noise disorganizes neural timing and accentuates interindividual differences in the representation of formant transitions, especially among individuals with poorer reading and poorer speech-in-noise perception, reinforcing that this vulnerability emerges under conditions of high temporal demand [59,62]. Together, these findings support the interpretation that the deficit in dyslexia is not uniformly low-level sensory in nature but reflects a failure in the context-dependent modulation of subcortical speech encoding, sensitive to signal predictability, the presence of background noise, and brainstem–cortex integration demands [53,63].

It should be noted that this profile of alterations in subcortical speech encoding across different conditions is consistent with the core deficit of dyslexia, which lies primarily in the phonological processing of language [64,65]—a skill that depends fundamentally on the ability to process and analyze speech sounds and that is crucial for the acquisition of written language, and which was also markedly impaired in both siblings. In this sense, the data from the present study provides an objective measure of how subcortical speech encoding is degraded in these participants, suggesting the need for a more detailed understanding of whether there is a correlation between deficits in the auditory measures and in phonological processing. Furthermore, the poorer performance observed in the presence of noise underscores the importance of guidance directed at schools regarding classroom seating for these children, since school learning tends to occur in the presence of background noise, which may further hinder this process in individuals with dyslexia.

An important point to be discussed is that the masking noises were matched in sound level (70 dB SPL; SNR +10 dB) and differed only in route; the difference between the ipsilateral and contralateral conditions favors an interpretation in terms of medial olivocochlear efferent modulation in the contralateral condition, which has previously been reported in poor readers and in individuals with speech-in-noise difficulties [66].

It should be noted, however, that in the present report, assessment of otoacoustic emissions with suppression—which could have helped analyze the contribution of the medial olivocochlear reflex to the FFR—was not performed. The absence of a physiological measure independent of MOC function limits the robustness of this interpretation. The inclusion of otoacoustic-emission suppression measures would have allowed a convergent analysis between peripheral cochlear mechanisms and central electrophysiological responses, strengthening the inference regarding the role of the efferent system in the subcortical encoding of speech in noise.

### 6.4. Cognitive Evoked Potential (P300)

The P300 cognitive potential was altered in both siblings, although with differences between their responses. In Sibling 1, the P300 component was absent in the left ear; in Sibling 2, prolongation of the P300b latency was observed in the right ear. These findings are consistent with the literature [67], which describes increased P300 latency in children with dyslexia in the auditory oddball paradigm, suggesting reduced allocation of attentional resources and slowed information processing. The mirrored configuration between siblings—normal findings on the right and alteration on the left in one, and the opposite pattern in the other—shows that the electrophysiological alteration does not follow a fixed pattern, even between siblings, reinforcing the interindividual heterogeneity [68] described in dyslexia.

It should be noted that this opposite lateralization is not supported by any expected physiological asymmetry. In typical listeners, monaural stimulation does not produce a significant difference in P300 latency or amplitude between the right and left ears [69], and P300 latency does not show a consistent hemispheric effect, although amplitude may show slight scalp asymmetry [70]. Thus, the crossed pattern observed here is not attributable to a normal interaural asymmetry, and should be interpreted, in this case study, as an expression of the interindividual variability of the phenomenon, rather than as evidence of a functional asymmetry mechanism—a hypothesis that would require a larger sample to be tested. A comparably similar interaural P300 asymmetry, with an absent response in one ear and prolonged latency in the other, has previously been documented in a case report involving an adolescent with comorbid Developmental Language Disorder and Specific Learning Disorder, reinforcing that this pattern may not be exclusive to dyslexia alone [71].

The P300 impairments are consistent with the deficits in attention, working memory, and executive functions that were also documented in the siblings’ neuropsychological assessment [34,35,36,40]. The prolonged latencies, amplitude alterations, and absence of responses may represent slowed processing that accompanies the phonological-processing difficulties. This also reinforces the interindividual heterogeneity of the dyslexic phenomenon: even between siblings sharing genetic background and diagnosis, the electrophysiological responses diverged [67]. Complexes such as MMN and P300 are identified with substantially lower frequency in dyslexic children than in controls, in about one-third of cases, and do not show the age-related maturation observed in typical readers [67], which relates both to the degree of impairment and to the variability of the condition. This set of alterations in central cognitive components, rather than in indices of peripheral sensory acuity, supports the conception of dyslexia as a neurodevelopmental disorder of predominantly central origin, in which the phonological deficit constitutes the most consistent and most heritable finding, while low-level sensory deficits show more limited prevalence and effect [72].

A complementary analysis, derived from the P300 responses themselves, examined behavioral performance grouped according to latency classification, without relying on an external normative reference. The two conditions with normal latency (Sibling 2’s left ear and Sibling 1’s right ear) accounted for the two highest hit rates in the sample (53.7% and 46.6%), higher than those of the conditions altered by prolongation (38.8%) or absence (43.4%). This correspondence internally corroborates the electrophysiological findings and is consistent with the established relationship between P3b latency and reaction time in tasks requiring stimulus categorization [35,41]. This correspondence, however, is clear for accuracy but only partial for response consistency: Sibling 1’s right ear, with normal latency, showed elevated variability (RMS deviation of 103 ms), indicating that reaction time, RMS deviation, and hit rate are not redundant measures with one another or with latency. It should also be noted that P300 abnormalities can coexist with preserved behavioral performance [65]; thus, the correspondence described here should be read as an exploratory, within-subject observation (reduced n per condition), rather than as a generalizable relationship.

It is worth noting that the electrophysiological measures corroborate the intra-familial heterogeneity: despite the shared diagnosis, kinship, and identical controlled data collection, the siblings differed in the mechanisms of impairment at each level assessed by the electrophysiological responses, which is consistent with the existence of subcortical subtypes [57] and with multifactorial/probabilistic models of dyslexia [10,73].

## 7. Limitations and Future Directions

This report is based on two subjects, which restricts any statistical inference and requires descriptive, exploratory reading. The opposite lateralization between siblings is interpreted here as heterogeneity; its possible association with functional hemispheric asymmetries or ear-specific audiological findings remains a hypothesis to be tested in a larger sample. Two normative gaps converge in this case and delimit future research agendas: the absence of a P300 latency norm for the 13-to-16-year age range, which Sibling 1 exemplifies, and the lack of norms for reaction time, RMS deviation, and hit rate in the oddball paradigm, whose lack of redundancy, suggested by the present data, could be formalized in studies with typical and atypical samples stratified by age. Related to these aspects, it should be noted that assessment of otoacoustic emissions with contralateral suppression was not performed, which restricts the direct interpretation of the contribution of the medial olivocochlear (MOC) efferent system to the FFR findings. Future studies should therefore integrate multimodal approaches to further elucidate the relationship between the FFR and auditory efferent function. Four further limitations should be acknowledged. First, general intellectual functioning was estimated with different Wechsler instruments (WASI for Sibling 1, WISC-IV for Sibling 2) for the clinical reasons described above; although both are normed for the Brazilian population, the resulting FSIQ values are not strictly comparable between siblings. Second, all components were identified by manual marking. Although every waveform was marked blind and independently by two experienced audiologists, with divergences adjudicated by a third and more experienced rater, agreement between the two independent raters was not quantified by a reliability index; this way, studies using this protocol in larger samples should report a formal agreement index such as the intraclass correlation coefficient.

## 8. Conclusions

In this report of two siblings with familial dyslexia, the multimodal electrophysiological battery revealed preserved brainstem sensory transmission (ABR), in contrast to noise-vulnerable speech encoding (FFR) and altered cortical cognitive processing (P300). The findings illustrate, at both the individual and family level, the central, biologically heterogeneous, and multifactorial nature of dyslexia, and support the combined use of ABR, FFR, and P300 as an objective and complementary characterization of auditory processing in the disorder.

## Figures and Tables

**Table 1:** Acquisition parameters for the auditory electrophysiological protocols (click-ABR, P300 and FFR): electrode montage, intensity, polarity, stimulus rate, number of sweeps, filters, equipment and transducers.

Procedure	Electrode Placement	Intensity	Polarity	Stimulus Rate	Sweeps	Filters	Equipment	Transducer
ABR	M1, M2, Fz	80 dB nHL	Rarefaction	19.3/s	2 × 2000	1–30 Hz	NeuroAudio^TM^—Neurosoft, Ivanovo, Russia	Insert (ER-3A)
P300	M1, M2, Cz, Fz	75 dB nHL	Alternating	1.1/s	1 × 300	1–30 Hz	NeuroAudio^TM^—Neurosoft, Ivanovo, Russia	Insert (ER-3A)
FFR	M1, M2, Cz	80 dB SPL	Alternating	10.9/s	2 × 3000	100–2000 Hz	Bio-logic, Navigator Pro ^TM^—Natus Medical, Middleton, WI, USA	Insert (ER-3A)

Legend: dB SPL = decibel sound pressure level; dB nHL = decibel normal hearing level; ; Hz = hertz.

**Table 2:** Click-evoked auditory brainstem response (ABR): absolute latencies (waves I, III, V), interpeak intervals (I–III, III–V, I–V) and V/I amplitude ratio in the two siblings, by ear (80 dB nHL), with normative reference (Sanfins et al., 2026) [38].

Subject	Ear	I (ms)	III (ms)	V (ms)	I–III (ms)	III–V (ms)	I–V (ms)	I–Ia (µV)	V–Va (µV)	V/I Amplitude Ratio
Sibling 1	Right	1.46	3.76	5.69	2.30	1.93	4.23	0.18	0.53	3.02
Sibling 1	Left	1.36	3.61	5.49	2.25	1.88	4.13	0.27	0.35	1.30
Sibling 2	Right	1.34	3.64	5.65	2.30	2.01	4.31	0.50	0.50	1.00
Sibling 2	Left	1.30	3.55	5.56	2.25	2.01	4.26	0.46	0.31	0.68
Δ (S1 – S2)	Right	+0.12	+0.12	+0.04	0.00	−0.08	−0.08	−0.32	+0.03	+2.02
Δ (S1 – S2)	Left	+0.06	+0.06	−0.07	0.00	−0.13	−0.13	−0.19	+0.04	+0.62
Normative ♂	Right	1.17–1.71	3.37–4.08	5.10–6.20	1.97–2.61	1.61–2.25	3.74–4.69	—	—	—
Normative ♂	Left	1.15–1.70	3.35–4.15	5.07–6.57	1.64–3.06	1.16–2.96	3.14–5.31	—	—	—

Legend: ms = milliseconds; µV = microvolts; Δ = Sibling 1 – Sibling 2 (same ear); Normative = Sanfins et al. (2026) [38] ♂ male. Note: all values are descriptive observations obtained from single recordings; no statistical comparison was performed and no inference about statistical significance is intended.

**Table 3:** Frequency-following response (FFR) to /da/: component latencies (V–O), V–A interval, V–A slope, sustained-region RMS and spectral magnitudes (F0, F1) in the two siblings across the three listening conditions (quiet, ipsilateral noise, contralateral noise).

Subject	Cond.	V (ms)	A (ms)	C (ms)	D (ms)	E (ms)	F (ms)	O (ms)	V–A (ms)	Slope V–A (µV/ms)	RMS (nV)	F0 (nV)	F1 (nV)
Sibling 1	Quiet	7.12	8.03	19.03	22.45	31.36	40.20	48.11	0.91	−0.30	72.5	74	15
Sibling 1	Ipsil.	8.53	10.62	20.53	26.28	34.61	43.53	50.70	2.09	−0.04	26.2	23	4
Sibling 1	Contral.	7.20	8.03	18.62	22.37	31.36	40.11	48.03	0.83	−0.27	63.7	72	13
Sibling 2	Quiet	6.45	8.28	17.95	22.28	30.78	39.20	48.11	1.83	−0.14	66.6	51	18
Sibling 2	Ipsil.	7.37	8.78	19.87	22.70	30.78	39.28	48.36	1.41	−0.05	44.1	35	12
Sibling 2	Contral.	7.53	9.53	20.45	22.95	32.11	39.53	47.95	2.00	−0.06	48.5	11	8

Legend: Cond. = condition; Ipsil. = ipsilateral; Contral. = contralateral; ms = milliseconds; µV = microvolts; RMS = root-mean-square; nV = nanovolts. Note: all values are descriptive observations obtained from single recordings; no statistical comparison was performed and no inference about statistical significance is intended.

**Table 4:** P300 (long-latency auditory event-related potential): component latencies versus McPherson (1996) [34] age norm, mean reaction time, response consistency (RMS deviation) and target-detection accuracy in the two siblings, by ear.

Subject	Ear	P300 Component	Latency (ms)	Normative 5–12 Years Old (241–396 ms)	Mean RT (ms)	RMS Deviation (ms)	Correct Targets (%)
Sibling 1	RE	P300b	391.6	Normal	367	103	46.6
Sibling 1	LE	—	absent	Absent	399	90.3	43.4
Sibling 2	RE	P300b	456.4	Prolonged	356	117	38.8
Sibling 2	LE	P300	371.7	Normal	355	88.9	53.7

Legend: ms = milliseconds; RE = right ear; LE = left ear; Mean RT = mean reaction time to target stimuli in ms. Note: Normative reference: McPherson DL, Late Potentials of the Auditory System (San Diego: Singular, 1996 [34]). Note: All values are descriptive observations obtained from single recordings; no statistical comparison was performed and no inference about statistical significance is intended.

## Data Availability

The raw data supporting the conclusions of this article will be made available by the authors upon request due to privacy concerns.

## Abbreviations

ABFW	Child Language Test (ABFW)
ABR	Auditory Brainstem Response
AEPASC II	Auditory-evoked potential analysis software
ASEBA	Achenbach System of Empirically Based Assessment
BRIEF	Behavior Rating Inventory of Executive Function
cABR	Complex (speech-evoked) ABR
CPT	Continuous Performance Test
CV	Coefficient of variation
dB HL	Decibels hearing level
dB SPL	Decibels sound pressure level
DC	Direct current (baseline offset)
DCDC2	Dyslexia susceptibility candidate gene
DOI	Digital Object Identifier
DSM-5-TR	Diagnostic and Statistical Manual of Mental Disorders, 5th ed., Text Revision
DYX1C1	Dyslexia susceptibility candidate gene
ER-3A	Insert earphones
F0	Fundamental frequency
F1	First formant
FFR	Frequency-Following Response
FSIQ	Full-Scale Intelligence Quotient
GWAS	Genome-Wide Association Study
HF	High-frequency region
Hz	Hertz
IES	Inverse Efficiency Score
IQ	Intelligence Quotient
KIAA0319	Dyslexia susceptibility candidate gene
MMN	Mismatch Negativity
MOC	Medial olivocochlear (efferent system)
ms	Milliseconds
MTA-SNAP-IV	Swanson, Nolan and Pelham Questionnaire (MTA version)
NeuroAudio	Evoked-potential recording system
nV	Nanovolts
OEA	Otoacoustic emissions
P300 (P3)	P300 event-related potential
P300a/P3a	Frontal P300 subcomponent
P300b/P3b	Parietal P300 subcomponent
RAN	Rapid Automatized Naming
RAVLT	Rey Auditory Verbal Learning Test
RMS	Root mean square
ROBO1	Dyslexia susceptibility candidate gene
RT	Reaction time
sFFR	Speech-evoked Frequency-Following Response
SNAP-IV	Swanson, Nolan and Pelham Questionnaire
SNR	Signal-to-noise ratio
TDE/TDE-II	School Achievement Test
µV	Microvolts
V/I	Wave V to wave I amplitude ratio
VA	V-to-A peak-to-trough interval/slope
V, A, C, D, E, F, O	FFR peak components to /da/ (onset–sustained–offset)
WASI	Wechsler Abbreviated Scale of Intelligence
WISC-IV	Wechsler Intelligence Scale for Children, 4th ed.
